# Comparison of automated and manual FISH for evaluation of *HER2* gene status on breast carcinoma core biopsies

**DOI:** 10.1186/1472-6890-13-13

**Published:** 2013-04-20

**Authors:** Christian Öhlschlegel, Doris Kradolfer, Margreth Hell, Wolfram Jochum

**Affiliations:** 1 Institute of Pathology, Kantonsspital St. Gallen, Rorschacher Strasse 95, CH-9007, St. Gallen, Switzerland

**Keywords:** Breast cancer, HER2, Automated FISH

## Abstract

**Background:**

Positive HER2 status identifies breast carcinomas that might respond to trastuzumab treatment. Manual *HER2* fluorescent *in situ* hybridisation (FISH) is the most readily used method to detect *HER2* gene amplification which defines positive HER2 status in addition to HER2 protein overexpression*.* Automation of *HER2* FISH may improve *HER2* gene testing. The aim of our study was to evaluate an automated *HER2* FISH assay for assessing the *HER2* genomic status.

**Methods:**

Core biopsies of 100 invasive breast carcinomas were analysed in parallel using the PathVysion™ HER-2 DNA Probe Kit and the Leica HER2 FISH System for BOND™. To assess inter-method agreement, concordance analysis was performed for various numerical and categorical *HER2*/CEP17 FISH parameters.

**Results:**

Carcinomas with all HER2 immunohistochemical scores were included (0+: 20; 1+: 20; 2+: 30; 3+: 30). Using either *HER2*/CEP17 ratio >2.2 or ≥2.0 as criterion for *HER2* amplification, high levels of concordance were observed between automated and manual FISH (concordance rate 96%, κ coefficient 0.92). High levels of inter-method agreement were also found for *HER2* copy number, CEP17 copy number, *HER2*/CEP17 ratio, the percentage of carcinoma cells with *HER2*/CEP17 ratio >2.2, and the presence of *HER2* genetic heterogeneity, *HER2* clusters and CEP17 polyploidy.

**Conclusions:**

*HER2* testing using automated FISH is feasible on breast carcinoma core biopsies. Automated *HER2* FISH using the Leica HER2 FISH System for BOND is a practical and efficient alternative to manual *HER2* FISH in evaluating the HER2 status of primary invasive breast carcinomas.

## Background

Trastuzumab treatment in patients with invasive breast cancer is based on the HER2 status of carcinoma cells [1]. According to ASCO/CAP recommendations, positive HER2 status is defined as uniform, intense membranous HER2 protein expression in >30% of tumour cells or *HER2* gene amplification in carcinoma cells [2]. Determination of HER2 status can be performed either by HER2 protein expression analysis using immunohistochemistry (IHC) or *HER2* gene copy number analysis. Manual fluorescent *in situ* hybridization (FISH) is the current most readily used method to determine the HER2 genomic status. Alternative *in-situ* methods such as silver-enhanced *in-situ* hybridization (SISH) and chromogenic *in-situ* hybridization (CISH) have been developed to detect *HER2* gene amplification [3]. For these bright field *HER2 in-situ* hybridization methods, manual and automated assays have demonstrated high overall agreement with manual *HER2* FISH [4-8].

The aim of this study was to evaluate a new fully automated *HER2* FISH assay, the Leica HER2 FISH System for BOND™, in comparison with manual *HER2* FISH using the PathVysion™ HER-2 DNA Probe Kit. We provide data that automated *HER2* FISH enables accurate assessment of *HER2* gene status on breast carcinoma core biopsies.

## Methods

### Patient tumour samples

A series of 100 invasive breast carcinoma core needle biopsy specimens were retrieved from the surgical pathology files of the Institute of Pathology, Kantonsspital St. Gallen. Patients were diagnosed between 2008 to 2010. Carcinomas were classified according to the WHO Classification of Tumours of the Breast (2012). The 100 cases comprised 91 invasive ductal carcinomas (91%) and 9 invasive lobular carcinomas (9%).

The study was approved by the local ethics committee (Ethikkommission St. Gallen).

### Histology

Haematoxylin-eosin (HE) staining was performed using standard histological techniques. HE-stained sections were used to select areas with invasive carcinoma for subsequent HER2 immunostaining and *HER2* FISH. Carcinoma *in situ* was excluded from the analysis. To correlate HER2 immunostaining and *HER2* FISH results at the cellular level, areas of interest were marked to compare manual and automated *HER2* FISH results from corresponding areas of serial sections of carcinoma core biopsies.

### HER2 immunohistochemistry

HER2 protein status of carcinoma cells was assessed using the Leica Bond™ Oracle™ HER2 IHC System (Leica Biosystems Newcastle Ltd, UK) on a Leica BOND-MAX™ instrument. HER2 protein expression was scored as 0+ (no staining), 1+ (weak and incomplete membrane staining), 2+ (strong, complete membrane staining in ≤30% of tumour cells or weak/moderate heterogeneous complete membrane staining in ≥10% of tumour cells), or 3+ (strong, complete, homogeneous membrane staining in >30% of tumour cells) [2]. Based on the HER2 immunohistochemical score, 100 carcinomas with scores 0+ (n = 20), 1+ (n = 20), 2+ (n = 30), and 3+ (n = 30) were selected for subsequent *HER2* FISH analysis.

### *HER2* FISH

Manual *HER2* FISH was performed using the PathVysion™ HER-2 DNA Probe Kit (Abbott Molecular Inc., Downers Grove, IL). For automated *HER2* FISH, the fully automated Leica HER2 FISH System for BOND™ (Leica Biosystems Newcastle Ltd, UK) was used on a Leica BOND MAX™ instrument. This test contains the same probes used in the manual PathVysion™ kit presented as ready to use dual *HER2* and chromosome 17 FISH probes, allowing simultaneous determination of both *HER2* and chromosome 17 copy number. The technique was performed as per the manufacturer’s instructions, using the default ‘FISH protocol A’ on the Leica BOND System. At least 60 invasive carcinoma cells in at least three randomly selected areas of invasive carcinoma were scored for nuclear *HER2* and chromosome 17 centromeric probe (CEP17) signals using a Leica DM6000B fluorescence microscope system (Leica Microsystems, Heerbrugg, Switzerland) equipped with a triple bandpass filter set (DAPI/Spectrum Green/Spectrum Orange). The FISH result for each carcinoma cell was recorded in a table, which was used to calculate the *HER2*/CEP17 ratio and to determine the frequency of tumour cells with a *HER2*/CEP17 ratio > 2.2. If the average *HER2*/CEP17 ratio of 60 carcinoma cells was 1.8 to 2.2 (equivocal), another 40 invasive carcinoma cells were scored, and the final ratio of the tumour was calculated from the 100 cells.

Using the ASCO/CAP recommendations [2], a tumour was classified as *HER2* nonamplified (*HER2*/CEP17 ratio < 1.8), equivocal (*HER2*/CEP17 ratio 1.8 - 2.2), or amplified (*HER2*/CEP17 ratio > 2.2). Two alternative definitions of positive *HER2* amplification status were also tested: mean number of *HER2* signals >6.0 in carcinoma cells and *HER2*/CEP17 ratio >2.0 [2]. *HER2*/CEP17 ratio >2.0 was used as it represents the evaluation criterion recommended by the manufacturer of the PathVysion™ HER-2 DNA Test. *HER2* genetic heterogeneity was defined as more than 5% but less than 50% of invasive tumour cells with a ratio higher than 2.2 [9]. The percentage of carcinoma cells with HER2 gene cluster (defined as more than 16 *HER2* signals per nucleus or the presence of not individually separable *HER2* signals) was also determined. *HER2* cluster positive carcinomas were defined by the presence of *HER2* gene clusters in ≥ 1% of tumour cells. For calculation of the *HER2*/CEP17 ratio, cells with *HER2* clusters were considered to have 16 HER2 signals. Chromosome 17 copy number changes were classified based on the mean number of CEP17 signals per cell according to Ma et al. [10]. Polysomy 17 was included in the analysis since it may interfere with calculation of the *HER2*/CEP17 ratio. A single pathologist blinded against the FISH results of the alternative assay and the results of the HER2 immunohistochemistry evaluated manual and automated FISH slides in a random order.

### Statistical analysis

Inter-method agreement was evaluated for the following *HER2* FISH parameters: *HER2* copy number, CEP17 copy number, *HER2*/CEP17 ratio, proportion of carcinoma cells with *HER2*/CEP17 ratio ≥ 2.2, *HER2* amplification status, *HER2* genetic heterogeneity, the presence of *HER2* gene clusters, and chromosome 17 polyploidy. Correlation analyses of categorical variables were performed using contingency table analysis, chi-square test (two-sided Fisher’s exact test), and calculation of concordance rates and Cohen’s κ coefficients. Pearson’s correlation coefficient and linear regression analysis were used to assess agreement of continuous variables. SPSS 16 for Windows (SPSS, Inc., Chicago, IL) was used. P values < 0.05 were regarded as significant.

## Results

### Concordance analysis for *HER2* amplification status between manual and automated *HER2* FISH

**Figure 1:**
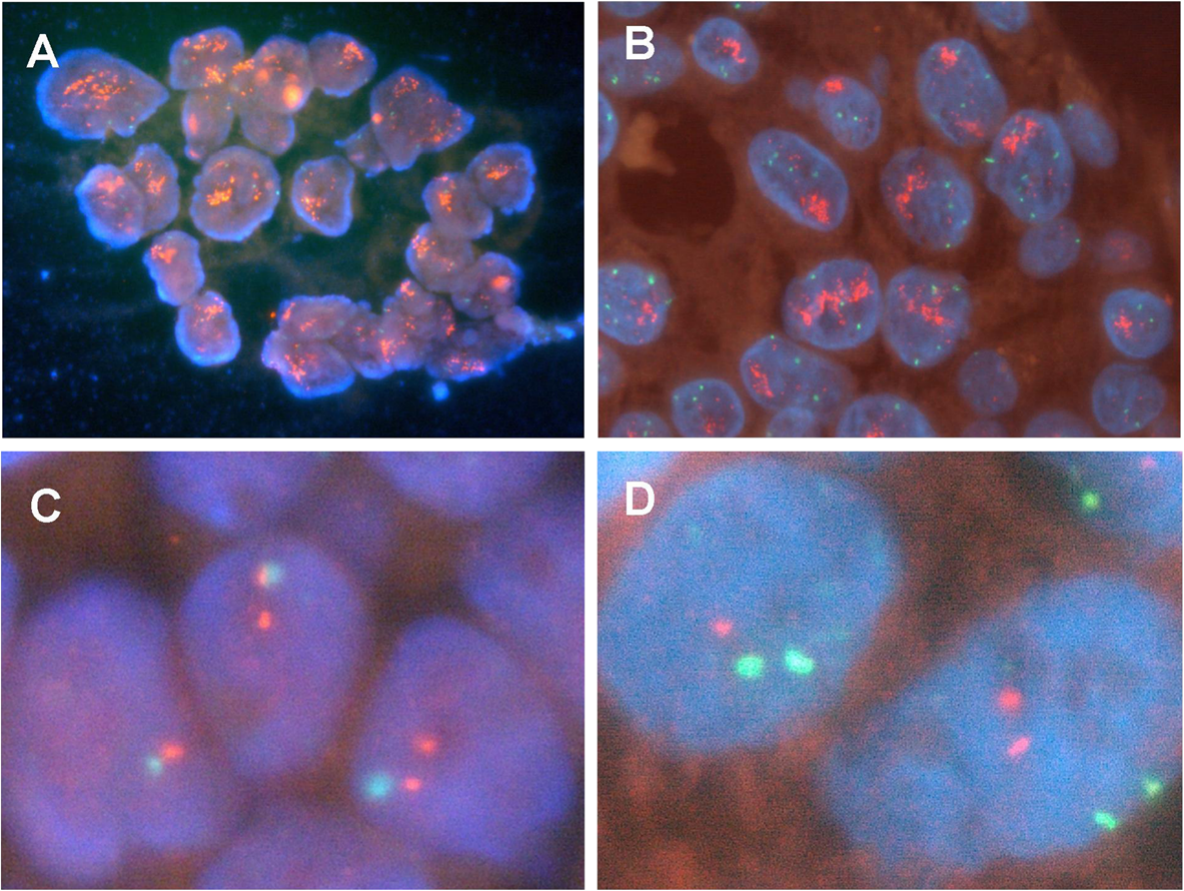
**Manual and automated *HER2/*CEP17 FISH.** Representative manual (**A**, **C**) and automated (**B**, **D**) *HER2*/CEP17 FISH results of carcinomas with (**A**, **B**) and without (**C**, **D**) *HER2* gene amplification. *HER2* signal red, CEP17 signal green. Original magnification 630 ×.

Both manual and automated *HER2* FISH were successfully performed on all samples (Figure 1). No *HER2* FISH failures were observed. Both manual and automated FISH produced bright and distinct signals for the *HER2* and CEP17 probes. Using ASCO/CAP scoring criteria for breast carcinoma, positive *HER2* gene amplification status is defined by a *HER2*/CEP17 ratio >2.2 in carcinoma cells [2]. Automated and manual FISH found *HER2* gene amplification in 46% and 50% of cases, respectively (Table 1). Four cases with *HER2* gene amplification according to manual FISH tested as not amplified by automated FISH. The overall concordance rate between manual and automated *HER2* FISH was 96% (K coefficient 0.920). Concordance rates were 100%, 100%, 93.3% and 93.3% for carcinomas with HER2 scores 0+, 1+, 2+ and 3+, respectively.

**Table 1:** **Concordance analysis between manual and automated *HER2 *FISH for *HER2 *amplification status**

**Parameter**	**Manual *HER2 *FISH**					**Automated *HER2 *FISH**						
	**0+ (n = 20)**	**1+ (n = 20)**	**2+ (n = 30)**	**3+ (n = 30)**	**Total**	**0+ (n = 20)**	**1+ (n = 20)**	**2+ (n = 30)**	**3+ (n = 30)**	**Total**	**Concordance rate (%)**	**Cohen’s kappa**
*HER2* amplification status (*HER2*/CEP17 ratio >2.2)												
Non-amplified (ratio < 1.8)	20 (100)	15 (75)	9 (30)	0	44 (44)	20 (100)	19 (95)	10 (33)	1 (3)	50 (50)		
Equivocal (ratio 1.8-2.2)	0	5 (25)	1 (3)	0	6 (6)	0	1 (5)	2 (7)	1 (3)	4 (4)	96 ^#^	0.920 ^#^
Amplified (ratio > 2.2)	0	0	20 (67)	30 (100)	50 (50)	0	0	18 (60)	28 (93)	46 (46)		
*HER2* amplification status (*HER2*/CEP17 ratio >2.0)												
Non-amplified (ratio < 2.0)	20 (100)	18 (90)	9 (30)	0	47 (47)	20 (100)	20 (100)	10 (33)	1 (3)	51 (51)	96	0.920
Amplified (ratio ≥ 2.0)	0	2 (10)	21 (70)	30 (100)	53 (53)	0	0	20 (67)	29 (97)	49 (49)		
*HER2* amplification status (Mean *HER2* number >6.0												
Non-amplified (number < 6.0)	20 (100)	20 (100)	12 (40)	3 (10)	55 (55)	20 (100)	20 (100)	14 (47)	4 (13)	58 (58)	97	0.939
Amplified (number >6.0)	0	0	18 (60)	27 (90)	45 (45)	0	0	16 (53)	26 (87)	42 (42)		

# Concordance rate and Cohen’s kappa value refer to the evaluation of amplified versus non-amplified *HER2* status. Equivocal *HER2* status (*HER2*/CEP 17 ratio 1.8-2.2) was considered as non-amplified for the calculation of concordance rate and Cohen’s kappa value.

In 4 cases, *HER2* amplification status (*HER2*/CEP17 ratio >2.2) was discordant between manual and automated FISH. *HER2*/CEP17 ratios of these cases were 2.62, 2.74, 2.5, and 3.14 for manual FISH, and 2.05, 2.08, 1.49, and 2.04 for automated FISH, respectively. Whereas all four cases were classified as *HER2* amplified based on manual FISH, 3 of these 4 cases were scored as equivocal and one case as not-amplified based on automated FISH. We therefore calculated mean *HER2* signal numbers, mean CEP17 signal numbers and mean *HER2*/CEP17 ratios of the study cohort for both manual and automated FISH. On average, manual FISH produced more *HER2* signals (Mean ± SE: 6.41 ± 0.46 versus 5.87 ± 0.42), but less CEP17 signals than automated FISH (Mean ± SE: 2.02 ± 0.04 versus 2.24 ± 0.06). The mean *HER2*/CEP17 ratio for automated FISH was smaller than for automated FISH (Mean ± SE: 2.56 ± 0.17 versus 3.13 ± 0.22). These results indicate that automated *HER2* FISH was associated with a trend towards lower *HER2*/CEP17 ratios and may underestimate the *HER2* amplification status of carcinomas with *HER2*/CEP17 ratios close to the cut-off value 2.2.

Using 2.0 as an alternative *HER2*/CEP17 cut-off value, *HER2* gene amplification was observed in 49% and 53% of carcinomas by automated and manual FISH, respectively (Table 1). The overall concordance rate between manual and automated *HER2* FISH was 96% (K coefficient 0.920). Concordance rates were 100%, 90%, 96.7% and 96.7% for carcinomas with HER2 immunohistochemical scores 0+, 1+, 2+ and 3+, respectively.

A third alternative definition of positive *HER2* amplification status is based on the mean absolute *HER2* gene copy number in carcinoma cells (average of >6 gene copies/nucleus) [2]. Using this definition, *HER2* gene amplification was observed in 42% and 45% of carcinomas by automated and manual FISH, with an overall concordance rate of 96% (K coefficient 0.939). Concordance rates were 100%, 100%, 93.3% and 96.7% for carcinomas with HER2 immunohistochemical scores 0+, 1+, 2+ and 3+, respectively.

Automated *HER2* FISH was also evaluated for its ability to identify carcinomas with positive HER2 status using HER2 3+ score as reference. Concordance rates between HER2 3+ score and positive *HER2* amplification status (*HER2*/CEP17 ratio >2.2) assessed by manual or automated *HER2* FISH were 100% and 97%, respectively.

### Concordance analysis for *HER2/*CEP17 FISH parameters between manual and automated *HER2* FISH

**Figure 2:**
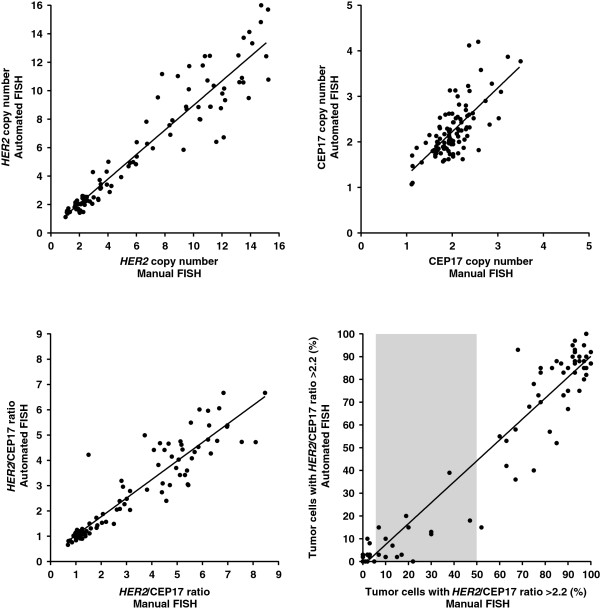
**Manual and automated *HER2 *testing in breast carcinoma.** Results obtained by manual and automated *HER2* testing using the same area of carcinoma are shown. Each carcinoma (n = 100) is depicted in the graphs according to the HER2 copy number (**A**), CEP17 copy number (**B**), *HER2*/CEP17 ratio (**C**) and proportion of carcinoma cells with HER2/CEP17 ratio >2.2 (**D**), respectively. Slope, intercept, and R square values of regression curves are summarized in Table 2.

Concordance analysis was also performed for four numerical *HER2*/CEP17 FISH parameters (Figure 2, Table 2). Manual and automated FISH results for *HER2* copy number, CEP17 copy number, *HER2*/CEP17 ratio and the percentage of carcinoma cells with *HER2*/CEP17 ratio >2.2 showed high correlation (Pearson correlation coefficient 0.947, 0.705, 0.943, and 0.970, respectively). Linear regression analysis revealed that *HER2* copy numbers, CEP17 copy numbers, *HER2*/CEP17 ratios and the percentages of carcinoma cells with *HER2*/CEP17 ratio >2.2 obtained by automated *HER2* FISH were highly consistent with the results of the manual assay (Table 2). Using Student’s *t* test for comparison, no significant differences between results of manual and automated FISH were observed for any of the four numerical *HER2*/CEP17 FISH parameters analysed.

**Table 2:** **Overall concordance analysis between manual and automated *HER2 *for four continuous *HER2*/CEP17 FISH parameters**

	**Manual *HER2 *FISH**	**Automated *HER2 *FISH**	**Correlation analysis**		**Linear regression analysis**		
Parameter	Mean ± SD	Mean ± SD	Pearson correlation coefficient	P value	Slope	Intercept	R square
*HER2* copy number	6.41 ± 4.6	5.87 ± 4.2	0.947	0.01	0.863	0.338	0.898
CEP17 copy number	2.02 ± 0.43	2.24 ± 0.59	0.705	0.01	0.962	0.299	0.497
*HER2*/CEP17 ratio	3.13 ± 2.19	2.56 ± 1.71	0.943	0.01	0.738	0.255	0.890
Proportion of carcinoma cells with *HER2*/CEP17 ratio >2.2 (%)	46.6 ± 41.6	41.1 ± 39.4	0.970	0.01	0.919	−1.732	0.942

*HER2* genetic heterogeneity, *HER2* clusters and CEP17 polysomy were identified in 17%, 41% and 4% of cases by manual FISH and in 14%, 39% and 12% of cases by automated FISH, respectively. Concordance rates between automated and manual *HER2* FISH results were high for all categorical *HER2*/CEP17 FISH parameters (Table 3).

**Table 3:** **Overall concordance analysis between manual and automated *HER2 *for three categorical *HER2*/CEP17 FISH parameters**

	**Manual *HER2 *FISH (%)**	**Automated *HER2 *FISH (%)**	**Pearson chi **^**2 **^**coefficient**	**P value**	**Concordance rate (%)**	**Cohen’s kappa**
*HER2* genetic heterogeneity						
Present (5-50% carcinoma cells with *HER2*/CEP17 ratio >2.2)	17	14	18.59	< 0.001	85	0.428
Absent	83	86				
*HER2* cluster						
Present (*HER2* cluster in >1% cells)	41	39	76.71	< 0.001	94	0.875
Absent	59	61				
CEP17 polyploidy						
Present (Chr. 17 copy number >3.00)	4	12	15.66	< 0.001	90	0.335
Absent	96	88				

Detailed comparison of manual and automated FISH results was also performed for carcinoma subgroups according to HER2 immunohistochemical score. Results for *HER2* copy number, CEP17 copy number, *HER2*/CEP17 ratio and the percentage of carcinoma cells with *HER2*/CEP17 ratio >2.2 were comparable between manual and automated FISH irrespective of HER2 immunohistochemical score (data not shown). No significant difference was observed for any of continuous *HER2*/CEP17 FISH parameters using Student’s *t* test. Within carcinoma subgroups according to HER2 immunohistochemical score, *HER2* genetic heterogeneity, *HER2* clusters and CEP17 polyploidy were detected with comparable relative frequencies using manual and automated *HER2* FISH (data not shown).

## Discussion

We compared automated with manual FISH using the same *HER2* and CEP17 probes in a large series of breast carcinoma core biopsies. Inter-method agreement was excellent for *HER2* amplification status using three different definitions (*HER2*/CEP17 ratio > 2.2, *HER2*/CEP17 ratio >2.0, mean *HER2* number >6.0) with overall concordance rates of 96%, 96% and 97%, respectively. Agreement was excellent over the complete range of HER2 immunohistochemical scores. We also observed high inter-method correlation for other categorical (*HER2* genetic heterogeneity, presence of *HER2* gene clusters and CEP17 polyploidy) and numerical variables (*HER2* copy number, CEP17 number and *HER2*/CEP17 ratio) between automated and manual FISH. Our results indicate that automated *HER2* FISH is a reliable method to determine the *HER2* gene amplification status in breast carcinomas and fulfills ASCO/CAP requirements for test validation of >95% concordance for amplified versus non-amplified cases [2].

Inter-method concordance analysis of biomarker assessment in FFPE breast carcinoma tissues can be negatively affected by various pre-analytical, analytical and post-analytical variables [2,11]. To limit variability we used core biopsies as material for our method comparison. Core biopsies are more homogeneous than resection specimens and tissue-microarray cores with regard to fixation conditions and more closely resemble the clinical situation in which *HER2* FISH is often performed on core biopsies, especially in patients in whom neo-adjuvant chemotherapy is considered. The HER2 status of invasive carcinoma cells was analysed by selecting areas of invasive carcinoma on haematoxylin-eosin stained sections. The reliability/concordance of the manual and automated FISH tests was assessed by comparing the results of automated and manual FISH obtained from the same carcinoma area. Scoring was performed by a single person experienced in FISH testing to reduce inter-observer variability. This approach closely resembles the process of HER2 status determination in routine diagnostics. Therefore, our results show that automated *HER2* FISH is a feasible and effective method for HER2 status determination in clinical practice.

In diagnostic pathology, the degree of automation is advanced for conventional and immunochemical staining methods. The potential advantages of total automation are decreased personnel and operating costs, less human intervention and fewer laboratory errors, more rapid processing of samples and recording of results, increased safety, better control of the entire process, and decreased need for laboratory space. In our study, we evaluated for the first time a fully automated *HER2* FISH assay. Similar to FISH, HER2 assays using silver-enhanced *in-situ* hybridization (SISH) or chromogenic *in-situ* hybridization (CISH) allow for simultaneous visualization of HER2 and CEP17 signals within the same carcinoma cell nucleus [3]. Various *HER2* bright field *in-situ* hybridization assays are available (e.g. Dako HER2 CISH pharmDX™ Kit, Ventana INFORM HER2 Dual ISH Assay, Zytovision ZytoDot 2C). Using manual *HER2* FISH as a reference and both histological and cytological material of various cancer types, automated bright field *HER2* assays have demonstrated comparable results suggesting that they represent reliable methods for *HER2* status determination [6,7,12-14]. Our results indicate that the same holds true for automated *HER2* FISH. Future studies may directly compare automated *HER2* FISH with automated single-colour or dual-colour CISH assays.

## Conclusion

In conclusion, our study indicates that automated *HER2*/CEP17 FISH is a reliable *in situ* method to analyse the *HER2* amplification status and in the future may represent a clinically useful tool for standardised and objective HER2 status evaluation of carcinoma cells, especially in combination with automated image analysis tools.

## Competing interests

Supported by Leica.

## Authors’ contributions

The study was planned by CO, DK, MH and WJ. Data acquisition was conducted by DK and CO. Data analysis was performed by WJ. WJ wrote the manuscript with help from CO. All authors read and approved the final manuscript.

## Pre-publication history

The pre-publication history for this paper can be accessed here:

http://www.biomedcentral.com/1472-6890/13/13/prepub
